# 

**Published:** 1911-10

**Authors:** 


VOL. XXVII. OCTOBER, 1911. No. 4

### TEXAS MEDICAL JOURNAL

Established July, 1885

*"Red Back"*

PUBLISHED AT AUSTIN, TEXAS, BY

F. E. DANIEL, M. D., - - - - Editor and Proprietor

PRINTED BY THE VON BOECKMANN-JONES [illegible]

**Subscription, $1.00 a Year, in Advance. - - - - Single Copies, 10 Cents**

Entered at the Postoffice at Austin, Texas, as second class mail matter.

